# Post Capsule Endoscopy Small Bowel Cancer Rate—An Australian Data Linkage Analysis

**DOI:** 10.1007/s12029-025-01313-w

**Published:** 2025-09-20

**Authors:** Paris Hoey, Naeman Goetz, Kimberley Ryan, Mark Appleyard, Florian Grimpen

**Affiliations:** 1 Department of Gastroenterology and Hepatology, Royal Brisbane and Women’s Hospital, Herston, 4029 Australia; 2 University of Queensland, Faculty of Medicine, Brisbane, Australia

**Keywords:** Small bowel, Capsule endoscopy, Cancer

## Abstract

**Purpose:**

Small bowel (SB) capsule endoscopy (CE) is a well-established investigation for suspected SB pathology, but little research has evaluated the diagnostic miss rate of SB cancer. This Australian study sought to assess the risk of developing SB cancer within 36 months of a cancer-negative CE (PCSBC-3Y) using a novel root-cause analysis (RCA) method.

**Methods:**

Using a prospective CE database and data linkage with a population-based state cancer registry, the PCSBC-3Y rate was evaluated in consecutive patients undergoing CE between 2007 and 2019. SB cancers diagnosed or suspected from CE were defined as ‘detected cancers’, whilst those diagnosed within 36 months of a negative CE were defined as ‘missed cancers’. Descriptive statistics summarised characteristics for all diagnosed SB cancers. All PCSBC-3Y cases were evaluated by RCA.

**Results:**

A total of 20 patients were diagnosed with SB cancer within 36 months of CE, comprising of 18 detected cancers and two missed cancers. The overall PCSBC-3Y rate was 10% (95% CI 2.6–28.7%). The two missed cancers included one jejunal gastrointestinal stromal tumour and one duodenal adenocarcinoma. RCA revealed both missed cancers to be unavoidable, as the SB was normal on two retrospective reviews of the CE video recordings.

**Conclusion:**

This study introduces the concept of measuring the PCSBC-3Y rate and a novel algorithm of RCA. In our quaternary referral CE cohort, the PCSBC-3Y rate was 10%. A subsequent RCA suggested that the two missed cancers were unavoidable.

**Supplementary Information:**

The online version contains supplementary material available at 10.1007/s12029-025-01313-w.

## Introduction

Small bowel (SB) cancer is rare. Its rarity and absence of specific clinical manifestations renders its diagnosis challenging and often delayed [1–5]. At the time of diagnosis, almost half of SB cancers have metastasised [1]. Most cases are unexpectedly diagnosed through diagnostic work-up of obscure or overt gastrointestinal bleeding that is due to ulceration of the tumour [2, 5].

Since its introduction into clinical practice in the early 2000s, SB capsule endoscopy (CE) has become an invaluable non-invasive diagnostic investigation for SB disease. It does not only have an excellent safety profile, but its potential to visualise the entire SB mucosa results in its high diagnostic yield [4, 6]. The diagnostic superiority of CE over conventional modalities (including push enteroscopy and small-bowel barium radiography) is well established, particularly for patients with obscure gastrointestinal bleeding [3, 4, 7]. As a result, international guidelines recommend CE as the first-line examination for suspected SB bleeding or SB tumours [8].

Despite high sensitivity for detecting lesions, CE has the potential to miss SB tumours that may be detected either on enteroscopy [9], repeat CE [10], or cross-sectional imaging [11]. Lesions arising from the duodenum or proximal jejunum, where rapid transit occurs, and subepithelial lesions with minimal endoluminal disruption (such as gastrointestinal stromal tumours and neuroendocrine tumours), are both risk factors for lesions being missed [5, 12]. Other limitations of detecting lesions include debris obscuring the camera lens, inadequate SB preparation, incomplete SB passage, and reader experience [11]. Despite these known limitations, there is a paucity of literature addressing the miss rate of SB cancer in CE.

This study aims to address this gap by evaluating the risk of developing SB cancer within 36 months of a cancer-negative CE, termed ‘post-capsule endoscopy SB cancer 3-year rate’ (PCSBC-3Y). The primary outcome was to quantify the PCSBC-3Y rate at an Australian quaternary centre. A secondary objective was to conduct root cause analysis (RCA) for each PCSBC case to identify the most plausible reasons for missed diagnoses.

## Methods

### Study Site

This is a single-site retrospective observational cohort study conducted at the Royal Brisbane and Women’s Hospital (RBWH), a quaternary referral centre in Brisbane, Australia. The gastroenterology unit currently undertakes about 280 CEs per year in a mix of local and regional adult patients. All CE are reported by nationally accredited gastroenterologists (MA and FG).

### Study Cohort

Data from all patients undergoing CE at our centre between 1 September 2007 and 31 December 2019 were extracted from Rapid Reader Electronic Endoscopic Reporting Software (Rapid Reader™ Software, Medtronic, Dublin – Ireland). This report was subsequently cross-referenced to the state-wide Queensland Cancer Register (QCR) to identify those patients who underwent CE and were also diagnosed with SB cancer between 1 September 2007 and 31 December 2022, such that every patient had at least 36 months of follow-up. It is a statutory requirement in Queensland, Australia, that all cancer diagnoses (except for keratinocyte skin cancers) are notified to the QCR, allowing complete acquisition of data for the study [13].

### Eligibility

All consecutive CE conducted in the study period were included for study review. Patients were excluded if the cancer diagnosis was diagnosed before or > 36 months after CE, and/or they had altered anatomy (e.g., gastrojejunostomy). In cases where several procedures were performed within 36 months preceding the diagnosis of SB cancer, only the most recent CE was included.

#### Study Data Definitions

A data dictionary was utilised to define all data variables and terms for the study. SB cancers diagnosed or suspected from CE were defined as ‘detected cancers’ (‘true positive’ CE), whilst those diagnosed within 36 months of a negative CE were defined as ‘missed cancers’, with the CE defined as a ‘false negative’ (FN). The PCSBC-3Y rate was calculated as *false negative-CE/(false negative* + *true positive-CE)*. Patient demographic details, including a personal history of hereditary polyposis or other cancer syndromes, as well as previous SB cancer(s), were collected. Comorbid conditions were translated into the Charlson Comorbidity Index (CCI) for each patient [14]. In addition, procedure-related data pertaining to the CE indication, quality of SB preparation, SB transit time (SBTT), endoscopic findings, and recommendations for further investigations were obtained. SB cancer-specific data included cancer location, histological type, grade, stage, and relevant tumour markers. Gastrointestinal lymphoma stage was described according to the Lugano lymphoma classification [15]; all other malignancy stages were in accordance with the criteria of the American Joint Committee on Cancer 8th edition [16].

As per hospital protocol, all patients are restricted to a clear fluid diet for 12 h followed by an overnight fast, prior to the CE appointment. A purgative bowel preparation is not used at our centre [17]. All patients receive simethicone shortly before swallowing the capsule. SB preparation scored as fair, good, or excellent was deemed adequate. SBTT was defined as the length of time between the first duodenal image and the first caecal image, and classified as a complete study if the caecum was reached. Time to diagnosis was defined as the median time (in days) from CE to diagnosis, as recorded by the QCR.

Medical records of all SB cancer cases were reviewed to ensure that they met the inclusion criteria and were appropriately categorised as detected or missed cancers. This involved one senior gastroenterologist (FG) carefully re-reviewing each CE report. The study team met regularly to discuss any queries, as well as the patient deaths in the study cohort, which were individually adjudicated by the gastroenterologist.

### Statistical Analysis

Descriptive statistics were utilised to summarise frequencies and percentages for categorical data, and median (interquartile range (IQR)) for non-normally distributed continuous data.

**Fig. 1:**
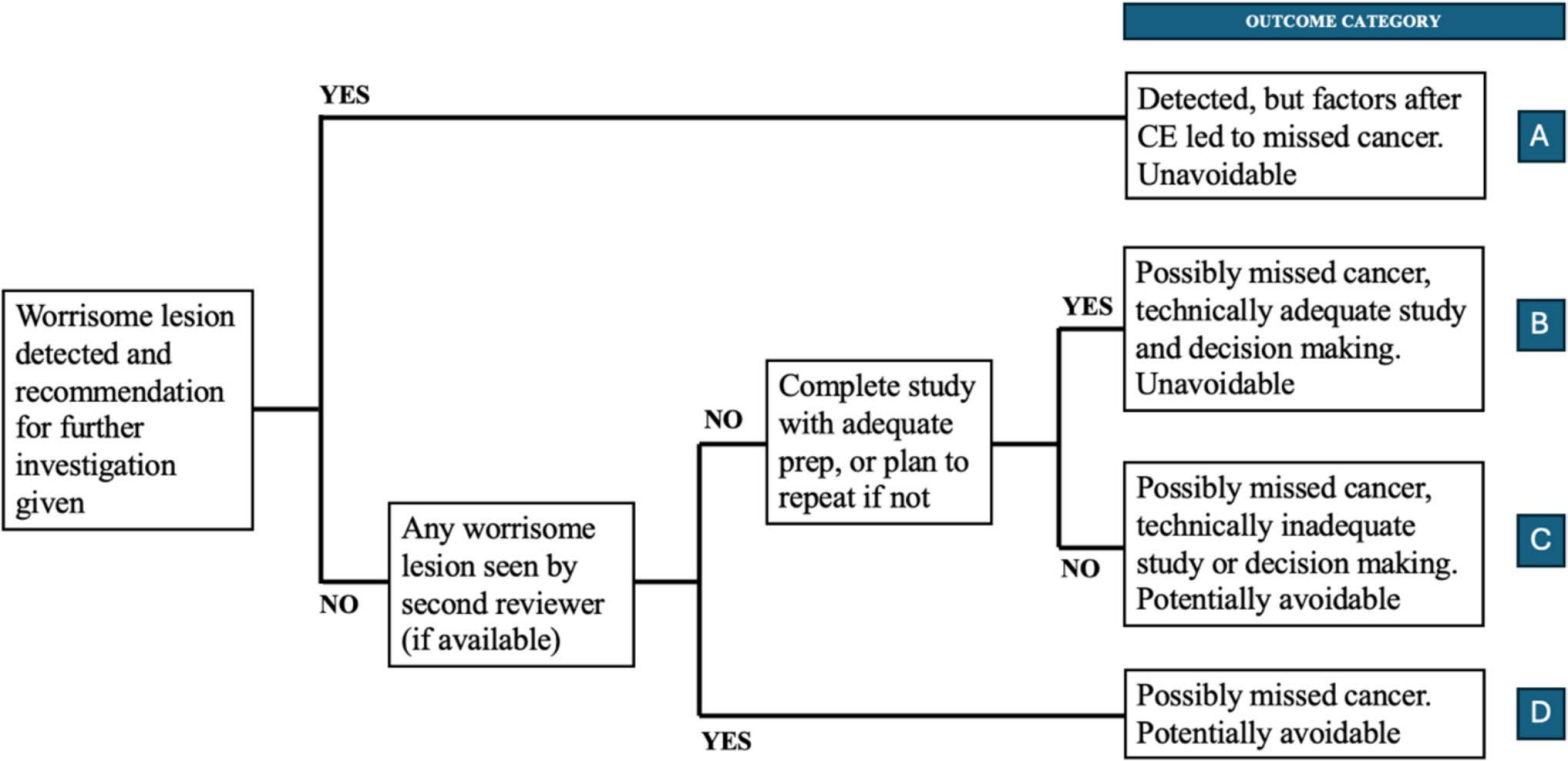
Our novel RCA system to determine most plausible PCSBC explanation

A novel system of RCA for PCSBC was devised as depicted in Fig. 1, which is analogous to published systems for missed cancers in upper and lower gastrointestinal endoscopy [18, 19]. All cases of missed cancer were retrospectively reviewed by two experts (FG and MA), who initially assessed whether a worrisome lesion had been detected on the original CE review and if recommendations for further investigation had been provided. Evaluation of the adequacy and completeness of the study, including careful review of the video recording, as well as the appropriateness of follow-up decisions, was subsequently undertaken. Based on these criteria, each case was classified into one of four outcome categories (A–D):

A) Detected lesion but factors after CE led to missed cancer; unavoidable.

*Example: Lesion detected on original review with a recommendation for retrograde enteroscopy but the patient was lost to follow-up.*

B) Possible missed lesion despite adequate examination and decision-making; unavoidable.

*Example: No lesion was detected on two expert reviews despite excellent SB preparation and complete transit.*

C) Possible missed lesion with technically inadequate study and/or decision-making; potentially avoidable.

*Example: No lesion was detected on two expert reviews; however, the SB preparation was poor precluding adequate visualisation and no repeat CE had been arranged.*

D) Possible missed lesion detected by the second expert, possibly due to inadequate video assessment; potentially avoidable.

*Example: No lesion was detected on original review but subsequently seen on second review.*

#### Ethical Approval

Ethics approval was granted for the study by the Metro North Health Human Research Ethics Committee (EC00172).

## Results

**Fig. 2:**
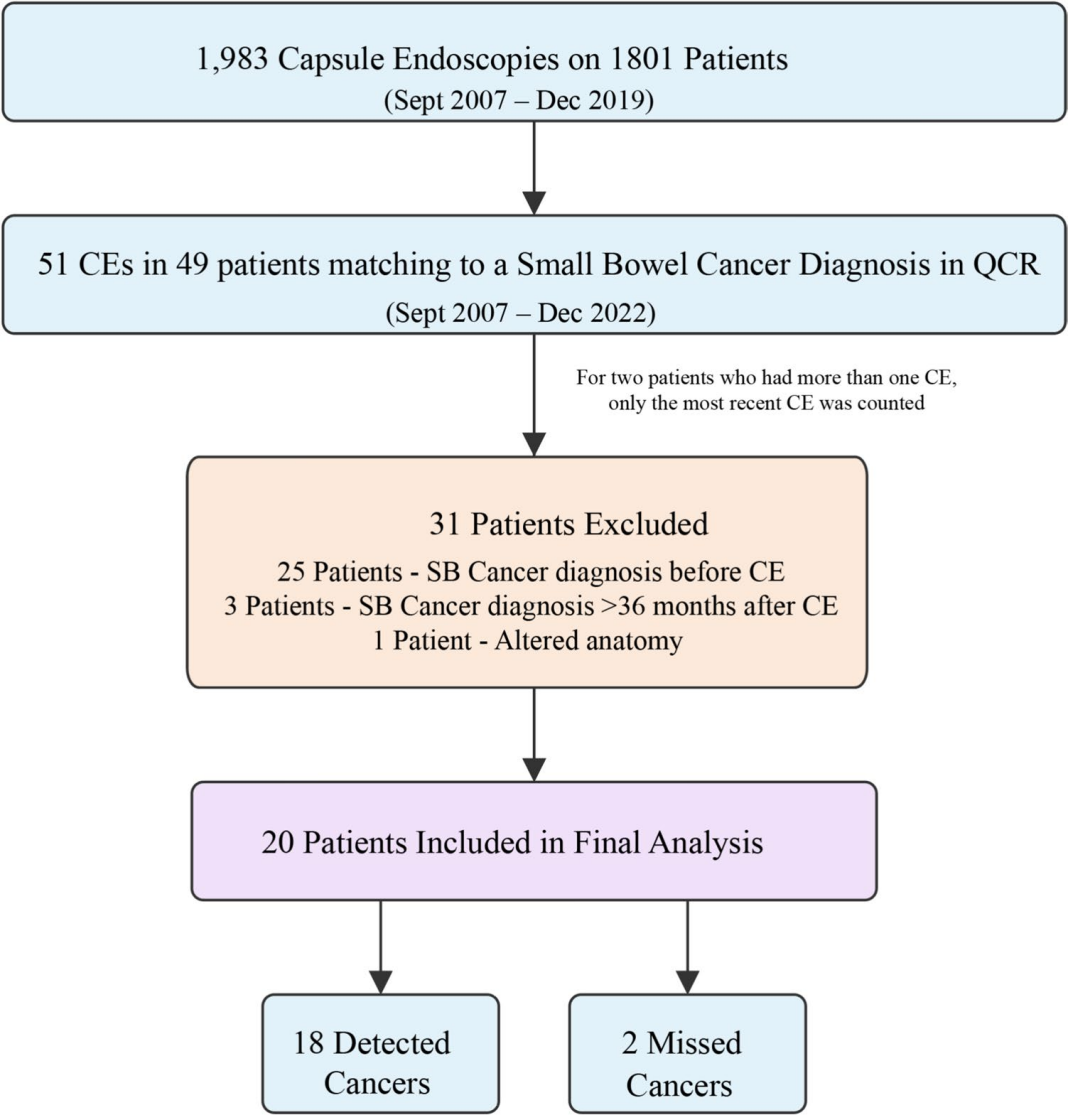
Patient selection

There were 1983 CEs performed on 1801 patients between September 2007 and December 2019. Of those, 49 patients were matched with the QCR as having an SB cancer diagnosis during the study recruitment and follow-up period (2007–2022). Following the application of exclusion criteria (Fig. 2), 20 patients were included in the final analysis, which comprised of 18 detected cancers and two missed cancers. The overall PCSBC-3Y rate was 10% (95% CI 2.6–28.7%). The incidence of SB cancer among patients undergoing CE was 1.0%.

Table 1 summarises the baseline characteristics of the study cohort. The median age at diagnosis was 69 years (IQR, 59–78) for detected cancers and 41 years (range 22–60) for missed cancers. The primary indication for CE in those with detected cancer included iron deficiency anaemia (IDA) (61%, *n* = 11/18), overt gastrointestinal bleeding (28%, *n* = 5/18), and abnormal imaging (11%, *n* = 2/18). Both patients with missed cancer had the CE to investigate IDA (100%, *n* = 2/2).

**Table 1:** Baseline characteristics of the study population, by cancer detection status

	**Detected cancers (*n* = 18)**	**Missed cancers (*n* = 2)**
**Age at diagnosis (years), median (IQR)**	69 (59–78)	41 (22–60)
**Male, *n* (%)**	14 (78%)	1 (50%)
**Previous SB cancer history**	1 (6%)^a^	0
**Hereditary polyposis syndrome**	0	0
**Other cancer syndrome**	0	1 (50%)^b^
**CCI, median (IQR)**	8 (5–9)	2.5 (2–3)
**CE indication, *n* (%)**		
Iron deficiency anaemia	11 (61%)	2 (100%)
Overt bleeding	5 (28%)	0 (0%)
Abnormal imaging	2 (11%)	0 (0%)
**CE characteristics**		
SB transit time (mins), median (IQR)	282 (200–312)	170 (166–174)
Complete SB passage, n (%)	15 (83%)	2 (100%)
Adequate preparation, n (%)	18 (100%)	2 (100%)
**SB cancer characteristics**^**c**^		
**Location, *n* (%)**		
Duodenum	1 (6%)	1 (50%)
Jejunum	5 (28%)	1 (50%)
Ileum	12 (67%)	0
**Histological subtype, *n* (%)**		
Adenocarcinoma	3 (17%)	1 (50%)
Gastrointestinal stromal tumour	3 (17%)	1 (50%)
Intestinal lymphoma	3 (17%)	0
Neuroendocrine tumour	9 (50%)	0
**Time to diagnosis (days), median (IQR)**	53 (13–222)	496 (446–546)

IQR, interquartile range; SB, small bowel; CCI, Charlson comorbidity index; CE, capsule endoscopy.

^**a**^This patient had a history of Hodgkin’s lymphoma in remission, and then was subsequently diagnosed with Stage IV, Grade 1 follicular (non-Hodgkin’s) lymphoma of small bowel involvement 4 years later.

^**b**^This patient had Lynch syndrome.

^**c**^See supplementary table 1 summarising the histological grade and stage of detected and missed cancers

In terms of SB cancer characteristics, most SB cancers in the detected cancer group originated from the ileum (67%, *n* = 12/18), followed by jejunum (28%, *n* = 5/18), with the most common histologic type being neuroendocrine tumour (NET; 50%, *n* = 9/18). The two missed cancers included one gastrointestinal stromal tumour (GIST) in the jejunum and one adenocarcinoma in the duodenum; both were early-stage cancers (stage I and IIa, respectively).

The median time to diagnosis of missed cancers was 496 days (range 446–546). Among the 9 patients with a SB NET (all in the detected cancer group), serum Chromogranin A and platelet serotonin tumour markers were elevated in 50% (*n* = 3/6) and 25% (*n* = 1/4) of cases with data available, respectively.

Seven patients died during the study period, all in the detected cancer group (38.9%, *n* = 7/18). There were no deaths in the missed cancer cohort. Three deaths were related to the consequence of metastatic disease from SB cancer, three deaths were unrelated, and one patient had an unknown cause of death. The three unrelated deaths occurred in patients who had a history of malignant SB tumour resection (with clear margins) and died due to comorbid-related conditions. There was inadequate information to determine the cause of death of one patient, and it was therefore listed as unknown.

The one patient with altered anatomy (Billroth II gastrojejunostomy) was excluded from the data (Fig. 2). Their CE, after negative bidirectional endoscopy, was performed to investigate IDA. The CE examination was incomplete with inadequate SB preparation, with a recommendation to consider further SB investigations if there was ongoing IDA. The patient re-presented 5 months later with vomiting, epigastric pain, and progressive weight loss. Cross-sectional imaging of the abdomen revealed an intraluminal polypoid mass of the oversewn proximal duodenal stump. Repeat endoscopy showed food remnants in the stomach and possible obstruction of the afferent loop. Following careful consideration of the risks and benefits of surgical resection, the patient chose not to pursue further investigations or intervention.

The 3-year interval for the PCSBC rate may not be as meaningful as with endoscopy or colonoscopy due to the slower growth rates of certain SB cancer (NET and GIST) compared to gastric and colon cancer from which the standardised 3-year rate originates (PCCRC-3Y and PEUGIC-3Y). We therefore performed a post-hoc analysis, increasing the interval to 5 years, with a corresponding reduction in the study recruitment period to 10 years to allow for this follow-up. This increase in follow-up had only a marginal effect on the results (PCSBC-5Y = 11.8% vs PCSBC-3Y 10%—data not shown).

### Root-Cause Analysis of PCSBC Cases

Table 2 provides data on the case details of the two patients who developed PCSBC. Both patients had CE to investigate IDA, following negative bidirectional endoscopy. Their studies had been deemed complete with adequate preparation, and there were no reported macroscopic abnormalities detected at the site of future SB cancer in 2/2 patients. Both CE video recordings were then reviewed by two experts (FG and MA), and no SB lesions could be identified. Therefore, the two patients were classified as having a possible missed lesion at CE, despite adequate examination and decision-making (unavoidable, Type B). Importantly, none of these missed cancers resulted in a death.

**Table 2:** Characteristics of two patients with PCSBC-3Y

Age and Sex	Cancer histological type, SB site, grade and stage	Details of false-negative CE	Complete CE study with adequate preparation	Further investigations recommended on CE report	Clinical details leading to SB cancer diagnosis	Time interval between FN-CE and diagnosis, months	Root cause analysis –PCSBC Type
22 M	GIST of the jejunum, G1, Stage I	**Indication:** IDA **Findings:** Normal small bowel	Yes **SBTT:** 174 min	No	Per rectal bleeding 2 years later with a bleeding SB lesion on CT-angiography	18	B (possible missed lesion, unavoidable)
60F Lynch syndrome	Adenocarcinoma of the duodenum, moderately differentiated, Stage IIA	**Indication:** IDA **Findings:** Normal small bowel	Yes **SBTT:** 166 min	No	Lesion identified 15 months later, arising from the scar of a previously resected duodenal low-grade adenoma	15	B (possible missed lesion, unavoidable)

PCSBC-3Y; post-capsule endoscopy small bowel cancer-3Y rate; SB, small bowel; CE, capsule endoscopy; FN-CE, false-negative capsule endoscopy; GIST, gastrointestinal stromal tumour; IDA, iron deficiency anaemia; SBTT; small bowel transit time; CT, computed tomography.

The first patient was a 22-year-old male who developed significant overt gastrointestinal bleeding 18 months after their negative CE for IDA. An urgent computed tomography angiography of the abdomen and pelvis showed active bleeding from the SB that was treated with surgical resection. Histology confirmed a GIST arising from the jejunum. Interestingly, the surgical specimen was described as an extrinsic mass arising from the jejunal wall, with intact overlying luminal mucosa. This highlights the previously documented reduced sensitivity of CE in identifying submucosal lesions [5, 12].

The other patient was a 60-year-old female with a history of Lynch syndrome and total colectomy with ileo-rectal anastomosis, who underwent CE to investigate mild IDA. Notably, this patient also had a history of a previously resected large low-grade duodenal adenoma. Prior to CE, bidirectional endoscopy was performed as a work-up for IDA, revealing a healthy-appearing scar at the duodenal endoscopic mucosal resection (EMR) site but otherwise a normal examination. The CE was performed 8 months after the duodenal adenoma resection and was reported as a normal SB examination. At surveillance gastroscopy 15 months later, there was a recurrence of the duodenal lesion with biopsies confirming moderately differentiated adenocarcinoma.

## Discussion

CE is an invaluable diagnostic modality for SB pathology, yet there is a paucity of literature on the miss rate of SB cancer in patients undergoing CE. There is only one study in the literature that has used a similar research design with data linkage to a population-based cancer registry to identify SB tumours. That study revealed a miss rate of 3 malignant SB tumours over a 6-year study period among 145 patients, with variable follow-up intervals [13]. In comparison, this present study was performed over 16 years and has included a study cohort of 1,801 patients. It is also the first study to determine the PCSBC-3Y rate and to subsequently apply a system of RCA specifically adapted for CE.

Previous studies have shown the CE miss rate of SB tumours to be between 16.5 and 18.9% [3, 20], compared to our centre’s PCSBC-3Y rate of 10%. However, these past studies included both benign and malignant lesions, while we included only malignant lesions. This may explain their higher rates, and makes comparison with the present study difficult. Similarly, neither of the past studies had a follow-up period, as they were both performed to primarily compare the diagnostic accuracy of the various modalities, including CE.

Our study utilised a 3-year interval rate to align with standardised post-colonoscopy colorectal cancer-3Y (PCCRC-3Y) and post-endoscopy upper gastrointestinal cancer-3Y (PEUGIC-3Y) rates. These may be used as quality metrics for endoscopy units, which allows for benchmarking clinical practice [18, 19, 21]. Changing the follow-up period from 3 to 5 years, to account for the slower growth rates of most small bowel tumours compared with those of the upper and lower gastrointestinal tracts, only marginally increased the miss rate in our study.

Utilising a RCA when assessing missed cancers may assist clinicians to identify contributing factors and may facilitate performance improvement in healthcare service delivery. Major international guidelines recommend assessment of several key performance measures of CE, which include: complete caecal or stomal visualisation, lesion detection rate, and capsule retention rate [22]. Currently, a PCSBC rate metric is not included. RCA for all cases with missed SB cancer was performed by a detailed medical chart review. The novel algorithm modified for CE from previously published RCA systems for missed cancers in upper and lower gastrointestinal endoscopy was followed [18, 19]. The two cases of missed cancers were deemed unavoidable, as a detailed review of the video recordings by two experts did not show any abnormality in the SB (Type B). Possible causes include submucosal or extrinsic growth of the lesion, the area of concern not being captured by the camera, and less commonly, rapidly progressive SB cancer arising de novo since CE. In the case of the patient with Lynch syndrome, it is probable that the cancer developed from a microscopic residual of the previously resected duodenal polyp.

In this study, the two missed cancers were of proximal SB origin. This is consistent with the published literature that suggests the rapid transit of the proximal SB to be a risk factor of missing lesions at these sites [3, 5, 12]. Similarly, one of the two missed cancers was a GIST, which corresponds to the well-documented challenge of detecting submucosal lesions [5, 12]. The scoring tools developed to assist in distinguishing between innocent mucosal bulging and submucosal lesions would not have been beneficial in this case, as no lesion was evident (SPICE & Shyung score) [23, 24].

### Limitations

This study has limitations which ought to be acknowledged. Firstly, despite nearly 2000 CEs being performed at the study site during the study period, the number of patients that were diagnosed with SB cancer was small, reflecting the low incidence of the disease (1.0%). This limited sample size resulted in wide confidence intervals for the PCSBC-3Y rate and precluded statistical analysis to identify factors predictive of PCSBC. Retrospective data collection is always fraught with weakness. For this study, the data may be subject to interpreter bias, a change in technology over time, and evolving reporting standards over the study period. However, the second expert review as part of the RCA process minimises the risk of interpreter bias for missed cancer cases. Additionally, not all PCSBC cases may have been captured by the statewide cancer registry due to potential patient re-location out of Queensland. Similarly, a small number of SB cancers detected at CE that did not have histological confirmation may not have been registered with the QCR. Although the QCR does include cancers diagnosed by clinical investigations only (without histology), this may not always occur. Metastatic SB disease from other primary cancers was also excluded from the QCR linkage. Therefore, the detected cancer cohort may be larger.

Important strengths of our study include a long study period, a large patient cohort assessed at a high-volume centre, and our comprehensive case ascertainment from a population-based cancer registry. Additionally, the incorporation of a novel RCA algorithm tailored for CE offers a unique methodological advancement to systematically evaluate missed cancers. Further multi-centre, prospective studies are needed to confirm these findings, identify risk factors for PCSBC, and refine quality metrics for CE services.

## Conclusion

In our Australian quaternary referral cohort, the PCSBC-3Y rate was 10%, with two missed cases of SB cancer. These were located in the proximal SB at sites deemed normal on preceding, high-quality CE studies. A standardized RCA algorithm tailored for CE may offer a structured approach to understanding and categorizing missed SB cancer. This study adds evidence to the diagnostic accuracy on capsule endoscopy.

## Supplementary Information

Below is the link to the electronic supplementary material. 

Supplementary file1 (12.6 KB)

## Data Availability

No datasets were generated or analysed during the current study.

## Abbreviations

CE: Capsule endoscopy
SB: Small bowel
PCSBC-3Y: Post-capsule endoscopy small bowel cancer 3-year rate
RCA: Root cause analysis
RBWH: Royal Brisbane and Women’s Hospital
QCR: Queensland Cancer Register
FN: False negative
CCI: Charlson Comorbidity Index
SBTT: Small bowel transit time
IQR: Interquartile range
CI: Confidence interval
IDA: Iron deficiency anaemia
NET: Neuroendocrine tumour
GIST: Gastrointestinal stromal tumour
EMR: Endoscopic mucosal resection
PCCRC-3Y: Post-colonoscopy colorectal cancer 3-year rate
PEUGIC-3Y: Post-endoscopy upper gastrointestinal cancer 3-year rate
