# Supplementary material for: Post Capsule Endoscopy Small Bowel Cancer Rate—An Australian Data Linkage Analysis
Source: J Gastrointest Cancer. 2025 Sep 20;56(1):190. doi: 10.1007/s12029-025-01313-w (PMC12450118; doi:10.1007/s12029-025-01313-w)
Supplement: Supplementary file 1 — Supplementary file1 (12.6 KB) [file 12029_2025_1313_MOESM1_ESM.docx]

**Supplementary appendix**

**Supplementary Table 1:** Table summarising histological grade and stage of detected and missed cancers. Gastrointestinal lymphoma stage was described according to the Lugano lymphoma classification; all other malignancy stages were in accordance with the criteria of the AJCC 8th edition, 2017.

| **Histological Type** | **Histological Grade** | **Stage** | **Detected Cancer** | **Missed Cancer** |
| --- | --- | --- | --- | --- |
| **NET** | Grade 1 | Stage I | 1 | 0 |
|  | Grade 1 | Stage II | 1 | 0 |
|  | Grade 1 | Stage III | 0 | 0 |
|  | Grade 1 | Stage IV | 1 | 0 |
|  | Grade 2 | Stage I | 0 | 0 |
|  | Grade 2 | Stage II | 1 | 0 |
|  | Grade 2 | Stage III | 1 | 0 |
|  | Grade 2 | Stage IV | 1 | 0 |
|  | Unknown | Stage II | 1 | 0 |
|  | Unknown | Unable to Determine | 2 | 0 |
| **Total NET** | | | **9** | **0** |
|  | | | | |
| **Adenocarcinoma** | Moderately Differentiated | Stage I | 0 | 0 |
|  | Moderately Differentiated | Stage IIA | 1 | 1 |
|  | Moderately Differentiated | Stage IIB | 2 | 0 |
| **Total Adenocarcinoma** |  | | **3** | **1** |
|  | | | | |
| **GIST** | G1 (Low Grade) | Stage I | 0 | 1 |
|  | G2 (High Grade) | Stage IIIA | 1 | 0 |
|  | G2 (High Grade) | Stage IIIB | 1 | 0 |
|  | Unknown | Unable to Determine | 1 | 0 |
| **Total GIST** |  | | **3** | **1** |
|  | | | | |
| **Lymphoma** | Grade 1 | Stage IV | 1 | 0 |
|  | Unknown | Stage II | 1 | 0 |
|  | Unknown | Stage IV | 1 | 0 |
| **Total Lymphoma** |  | | **3** | **0** |
